# Early Diagnosis of Autism Spectrum Disorder: A Review and Analysis of the Risks and Benefits

**DOI:** 10.7759/cureus.43226

**Published:** 2023-08-09

**Authors:** Chiugo Okoye, Chidi M Obialo-Ibeawuchi, Omobolanle A Obajeun, Sarosh Sarwar, Christine Tawfik, Madeeha Subhan Waleed, Asad Ullah Wasim, Iman Mohamoud, Adebola Y Afolayan, Rheiner N Mbaezue

**Affiliations:** 1 Internal Medicine, California Institute of Behavioral Neurosciences & Psychology, Fairfield, USA; 2 Health Education and Promotion, Walden University, Minnesota, USA; 3 Paediatrics, Al Zahra Private Hospital Dubai, Dubai, ARE; 4 Medicine and Surgery, Fazaia Medical College, Islamabad, PAK; 5 Pediatrics and Neonatology, October 6 University, Giza Governorate, EGY; 6 Internal Medicine, Lower Bucks Hospital, Bristol, USA; 7 Internal Medicine, Air University, Islamabad, PAK; 8 Internal Medicine, Fazaia Medical College, Islamabad, PAK; 9 Internal Medicine, University College of Medical Sciences, New York City, USA; 10 Health, Department of Health, Cape Town, ZAF

**Keywords:** ethical considerations, screening tools, treatment, risks, benefits, diagnosis, asd

## Abstract

Autism spectrum disorder (ASD) is a neurodevelopmental condition made up of enduring challenges in social communication and interaction and the presence of repetitive and restricted behavior patterns. Early diagnosis of autism is crucial for timely intervention and improved long-term outcomes. This review aims to explore some of its signs and symptoms, look into some diagnostic tools, and analyze the benefits and risks associated with an early diagnosis of autism.

The symptoms of ASD vary from child to child, some of which are: avoidance of eye contact, lack of response to names, excessive fear, and lack of interactive and pretend play. Early identification of these symptoms by caregivers and healthcare providers facilitates the need for diagnosis and appropriate interventions. Some screening and diagnostic tools that have been found to help make the diagnosis are the Modified Checklist for Autism in Toddlers, Revised with Follow-Up (M-CHAT-R/F), the Social Communication Questionnaire (SCQ), the Parents' Evaluation of Developmental Status (PEDS), and the Childhood Autism Rating Scale (CARS), amongst others.

The benefits of early diagnosis include the opportunity for early intervention, which has been shown to enhance developmental outcomes and improve adaptive skills. Early identification allows for the implementation of specialized interventions tailored to the specific needs of individuals with autism, targeting social communication, language development, and behavioral challenges. Furthermore, early diagnosis enables families to access appropriate support services, educational resources, and community programs, facilitating better coping mechanisms, reducing parental stress, and increasing adult independence.

However, early diagnosis of autism also entails certain risks. One significant concern is the potential for labeling and stigmatization, which can impact the child's self-esteem and social interactions. There is a risk of overdiagnosis or misdiagnosis, leading to unnecessary interventions and treatments. Additionally, the diagnostic process can be lengthy, complex, and emotionally challenging for families, requiring comprehensive assessments by multidisciplinary teams.

This review highlights the importance of a balanced approach when considering the benefits and risks of early diagnosis. Early identification allows for timely interventions that significantly improve developmental outcomes and quality of life for individuals with autism. To mitigate the risks, it is crucial to ensure accurate and reliable diagnostic procedures, support families throughout the process, and promote societal awareness and acceptance. We also highlighted some future directions in the management of autism, including the use of biomarkers and the use of artificial intelligence and learning for diagnosing ASD.

## Introduction and background

Autism spectrum disorder (ASD) is a neurodevelopmental condition that affects how an individual perceives and engages with others, leading to difficulties in social interaction and communication [1]. According to the Centers for Disease Control and Prevention (CDC), ASD is a neurodevelopmental disability attributed to brain differences [2]. Individuals with ASD frequently encounter social, communication, and interaction challenges and exhibit restricted or repetitive behaviors and interests. Additionally, people with ASD may demonstrate distinct approaches to learning, movement, and attention [2]. Autism is more common in males and children born prematurely and is strongly linked to the genetic disorder Fragile X [3].

Autism spectrum disorder is usually diagnosed in early childhood between the ages of 18 and 24 months, and over the years, there has been an increase in its prevalence. The global number of old and new cases of ASD has increased from 0.62% in 2012 to 1.0% in 2021 [4]. Of the childhood population in Mexico, 0.87% have been diagnosed with this disorder, whereas 1% have been diagnosed in South Thames, UK [5]. Its prevalence has been increasing over the last few years, and one in 45 children in the US is born with it [6]. The only study that explored the prevalence of autism in Sri Lanka [7] found that red-flag signs of autism, as specified by the American Academy of Neurology and Child Neurology Society, were present in 7.4% and that one in 93 children (1.07%) aged 18 to 14 months was diagnosed with autism. The increasing numbers could be due to the inclusion and diagnostic criteria modifying over time to be more inclusive and wider, overdiagnosis, or increased risk factors associated with the disorder itself [8]. The diagnosis of autism increased by 57% between 2002 and 2006 [9]. Compared with people without ASD, individuals with ASD have higher rates of depression (20% vs. 7%), anxiety (11% vs. 5%), sleep difficulties (13% vs. 5%), and epilepsy (21% with co-occurring intellectual disability vs. 0.8%) [10].

Interventions for autistic children consume many resources and facilities, mainly due to the need for a larger therapist/client ratio, as each autistic child needs an individualized set of interventions. To validate the amount of resources channeled to the management of autistic children, there needs to be adequate awareness of the disorder. Individuals must be well informed because family members of autistic children undergo significant financial and mental burdens, and the more uninformed they are, the greater the risk of misdiagnoses, thus making their child more complex and resistant to therapy [8].

## Review

Signs, symptoms, and ethical considerations of ASD

According to the Centers for Disease Control and Prevention (CDC), early signs can be observed from receptiveness to social interaction. Some indications of these behaviors include evading or lacking sustained eye contact, not responding to their name by the time they reach nine months of age, and a lack of display of facial expressions such as happiness, sadness, anger, and surprise. By the age of 12 months, participation in simple interactive games like pat-a-cake is absent. At the same age, gestures, such as waving goodbye, are limited. By 15 months, the child fails to share interests with others, such as demonstrating enthusiasm for objects they like. By 18 months, there is no pointing to indicate something interesting. By 24 months, the child does not show awareness or empathy when others are hurt or upset. By 36 months, there is no observation or inclination to join other children in play. By 48 months, there is no engagement in pretend play, such as assuming roles like a teacher or superhero. Lastly, by 60 months, there will be no singing, dancing, or performing activities. The restrictive or repetitive behaviors manifest in particular interest, such as arranging objects in a specific order and becoming upset when that order is altered. There is also the repetition of words or phrases, known as echolalia. Other repetitive behaviors include playing with toys, in the same manner, each time, displaying heightened focus on specific parts of objects (like wheels), reacting strongly to minor changes, harboring obsessive interests, adhering strictly to certain routines, engaging in hand-flapping, body rocking, or self-spinning motions, and demonstrating peculiar responses to sensory stimuli such as sound, smell, taste, appearance, or texture. As a consequence of the above early signs, an ASD patient can experience various issues ranging from gastrointestinal issues to seizures, anxiety, hyperactivity or inattentive behaviors, language disorder, and delayed cognitive development or learning skills [9].

Neurological differences, including ASD, are the inherent idea of neurodiversity. There have been questions about the descriptive appendage of ASD as a disease (a medical condition that impairs the normal functioning of an organism's body, leading to symptoms and signs of the illness, which can be caused by pathogens such as bacteria, viruses, fungi, and parasites, or by non-infectious factors like genetic mutations, lifestyle choices, environmental exposures, or autoimmune responses) or if it could be seen as a different kind of normal. This can be a complex and awkward stance for a pediatrician when the Diagnostic and Statistical Manual of Mental Disorders, Fifth Edition (DSM-V) categorizes autism as a disorder [10]. As many pediatricians have tried to propagate this as not being a disease, families of individuals with ASD often feel frustrated by this perspective. They may experience a sense of hopelessness when confronted with the challenges of caring for their loved ones, who may have difficulty expressing emotions or forming typical social connections. Neurodiversity for ASD presents itself with a complex ethical dilemma, recognizing that autism is not a disease and is also painfully draining for families. Still, the total does not foreclose treatment if it is medically appropriate. Medical professionals should be mindful of the goals of their interventions and continue to respect the personhood of people with autism when treatment options are considered [10].

Autism invokes more child stigmatization than other developmental diagnostic labels (e.g., developmental language delay). Parents need early support and reassurance to love their children. This might be the best way to create a healthy partnership and promote parental acceptance.

Benefits of an early diagnosis of ASD 

Receiving an early diagnosis (between the ages of two and five) can provide opportunities for therapies that could aid in developing specific areas in a young child, such as communication, social interaction, and movement skills. Opting for therapy at a young age could reduce the child's frustration and potentially enhance their quality of life. Since a child's brain is still growing during the early stages of life, early intervention may have a more significant impact than starting therapy later.

The benefits of early diagnosis of ASD have been supported by several studies, including a systematic review and meta-analysis by Vivanti et al., which found that early interventions led to significant improvements in cognitive, language, and social-emotional functioning in children with ASD [11]. Early diagnosis of ASD leads to earlier interventions, which have been shown to improve developmental outcomes in children with ASD. Parents who received an early diagnosis of their child's ASD were reported by Grzadzinski et al. to have lower levels of stress and anxiety [12]; therefore, they were more likely to access appropriate services and support for their child, as well as help reduce parental stress and improve family functioning.

Better social outcomes and greater independence in adulthood have been observed in children diagnosed early and having early intervention [13], saving healthcare costs and reducing stress for the family in the long run. The significant cost savings over the long term also reduced the need for more intensive interventions and special education services [14]. Early diagnosis has also been shown to lead to early entry into specialized educational programs tailored to the unique needs of children with ASD. These programs have improved academic or educational outcomes and increased socialization opportunities for children with ASD [15].

Overall, these studies support the importance of early diagnosis of ASD in improving outcomes for affected individuals and their families and reducing long-term healthcare costs.

Risks of an early diagnosis of autism spectrum disorder

Getting an early diagnosis of ASD has been associated with some uncertainties and negative outcomes, including overdiagnosis and overtreatment. Brookman-Frazee et al. reported that children who received an early diagnosis of ASD were more likely to receive medication and behavioral therapies, even if they did not meet the diagnostic criteria for ASD [16]. It has also been found that screening tools designed to identify ASD in toddlers have high false-positive rates, resulting in many children being referred for further assessment who do not ultimately receive an ASD diagnosis [17]. Carbone et al., in their study, found that parents of children with ASD who received an early diagnosis reported feeling stigmatized by others and expressed concerns about the potential negative impact of the label on their child's future opportunities and quality of life [18]. There are reports of high levels of stress and anxiety, exacerbated by challenges accessing and navigating appropriate services and support [19]. An early diagnosis of ASD may also lead to a delayed diagnosis of other conditions that may be present in the child, such as anxiety or attention deficit hyperactivity disorder (ADHD). In 2013, a study published by Sprenger L et al. uncovered that children who were diagnosed with ASD at an early stage were less inclined to receive a diagnosis of ADHD, despite displaying symptoms characteristic of ADHD [20], and this could be detrimental to their receiving appropriate management.

Screening tools and diagnostic methods for ASD

Autism spectrum disorder can sometimes be identified at 18 months or younger, but by age two, a diagnosis by an experienced professional can be regarded as reliable. Because there is no medical test, such as a blood test, to diagnose the disorder, doctors must look at the child's developmental history and behavior to make the diagnosis. The National Center on Birth Defects and Developmental Disabilities (NCBDD) recommends that children undergo screenings at nine months, 18 months, and 24 or 30 months. The American Association of Pediatrics (AAP) proposes that autism screening be included in standard 18- and 24-month checkups. Although most physicians adhere to both sets of guidelines, it is recommended that parents also take an active role [21].

Understanding children’s developmental milestones is critical to ASD diagnosis. The CDC's developmental milestones are guidelines for parents and healthcare providers to help track a child's development from birth to five years old. These milestones cover social and emotional skills, language and communication, cognitive development, and physical growth. The guidelines provide information on what children should be able to do at certain ages and can help identify potential developmental delays or concerns. It is important to note that children develop at their own pace, and the milestones should be used as a general guide rather than a strict rule [22]. Screening results do not provide a definitive diagnosis; they may indicate the need for a formal developmental evaluation. During this process, a specialist can check if the child meets the ASD diagnosis criteria [23].

Some commonly used screening tools are:

The Modified Checklist for Autism in Toddlers, Revised with Follow-Up (M-CHAT-R/F), is a commonly used screening tool for ASD in children aged 16 to 30 months. It is a 20-item questionnaire that can be completed by parents or caregivers and has good sensitivity and specificity [24].

A general developmental screening tool called the Ages and Stages Questionnaire (ASQ) looks at developmental difficulties at particular ages [25].

The Screening Tool for Autism in Toddlers (STAT) is specifically intended for utilization by community service providers who engage with young children in assessment or intervention contexts and possess knowledge in the field of autism. This screening tool comprises 12 items and typically requires approximately 20 minutes to administer. Activities such as imitation, play, requesting, and directing attention are used to evaluate important social and communicative behaviors [26].

The Social Communication Questionnaire (SCQ), consisting of 40 yes-or-no questions the child’s caregiver must answer, is a tool used to screen for ASD in children between the ages of four and 40 [27].

**Figure 1:**
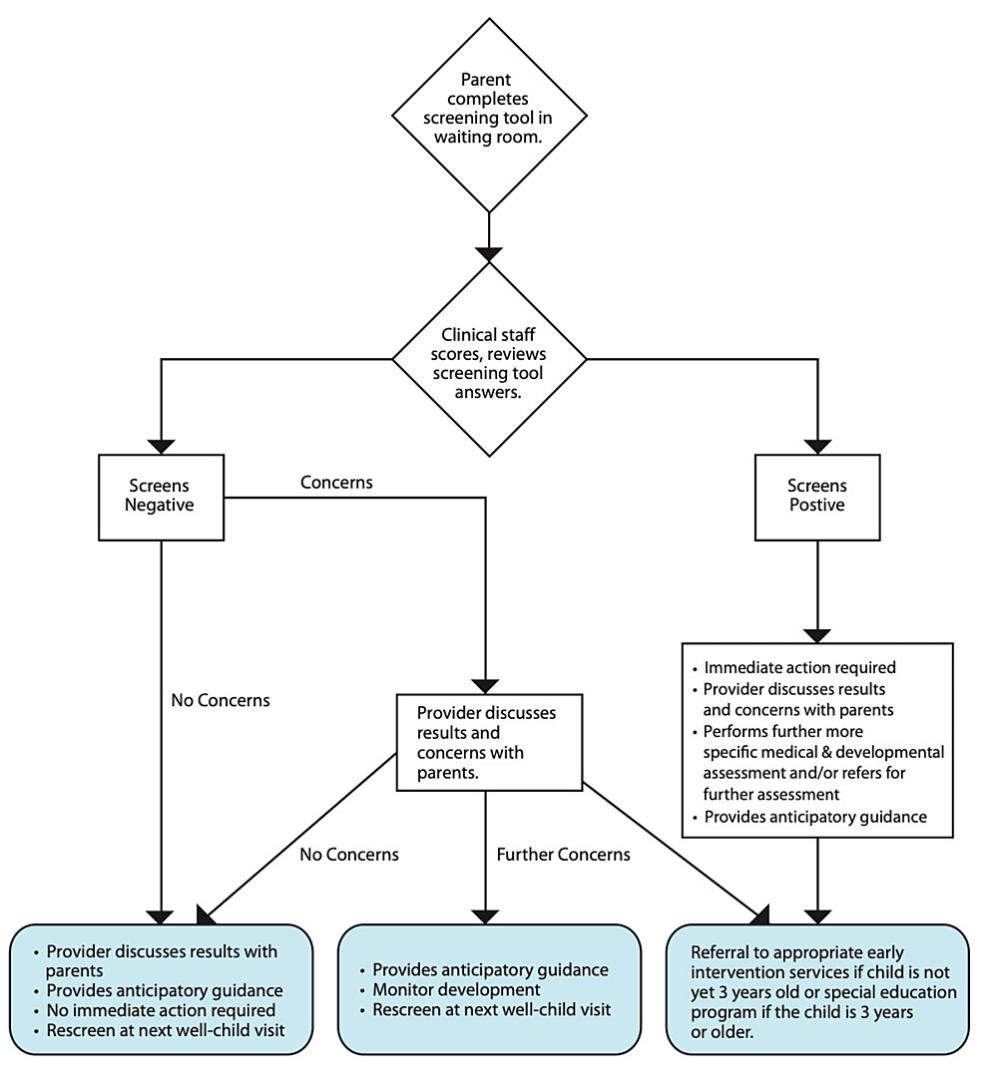
Pediatric developmental screening flowchart

The Parents' Evaluation of Developmental Status (PEDS) is a parent interview used to assess a child’s overall development in areas such as motor skills, language, and self-help, among others, to identify potential delays [25]. If the autism screening results suggest that your child exhibits certain autism-related behaviors, your pediatrician will consult a specialist for a thorough assessment. It is important to note that only a specialist (such as a child psychiatrist or psychologist, pediatric neurologist, or developmental pediatrician) has the expertise to provide an official diagnosis of autism [25]. Below in Figure 1 is a screening flowchart pediatricians use [28].

Some diagnostic tools used are the Autism Diagnosis Interview-Revised (ADI-R), a clinical diagnostic tool for evaluating autism in children and adults. The ADI-R is suitable for children and adults with mental ages of about 18 months and above. It focuses on behavior in three main areas: reciprocal social interaction; communication and language; and restricted and repetitive, stereotyped interests and behaviors. The Childhood Autism Rating Scale (CARS) is a concise evaluation tool for children aged two years and older. The CARS are based on five well-known diagnostic systems for ASD, and each item on the assessment addresses a specific trait, skill, or behavior of the child. Another diagnostic tool used is the Gilliam Autism Rating Scale, Second Edition (GARS-2), which supports clinicians, parents, and educators in recognizing and diagnosing ASD in individuals between the ages of three and 22. Additionally, it can assist in gauging the extent or intensity of the child's condition [28].

The Autism Diagnostic Observation Schedule-Generic (ADOS-G) is a formal assessment that follows a semi-structured and standardized approach to evaluate social interaction, communication skills, play, and creativity using materials for individuals suspected of having ASD. The evaluation is based on observing the individual's behavior and consists of four modules, each lasting 30 minutes. The modules are tailored to the individual's expressive language level [28].

The DSM-V offers a uniform set of standards that aid in identifying and diagnosing ASD, as shown in Table 1 below [29].

**Table 1:** DSM-V criteria for diagnosing autism spectrum disorder DSM-V: Diagnostic and Statistical Manual of Mental Disorders, Fifth Edition

Symptom criteria	Traits
Deficits in social communication and social interaction across multiple contexts	Deficits in social-emotional reciprocity; deficits in nonverbal communication behaviors (e.g., gestures) used for social interaction; deficits in the development, maintenance, and comprehension of relationships
Restricted, repetitive patterns of behaviors, interests, or activities	Stereotyped movements, speech, and use of objects; inflexibility to routine change or ritualized patterns; restricted interests with strong, abnormal attachments; hyposensitivity or hypersensitivity to environmental factors
Symptoms must be present in the early developmental period	Symptoms may not manifest until social demands exceed limited capacities
A combination of symptoms that significantly impair daily functioning	Level 3: requiring very substantial support; Level 2: requiring substantial support; Level 1: requiring support

Treatment options for autism spectrum disorder 

Autism spectrum disorder is not a disease, but a disorder. Therefore, treatments seek to slow down symptoms that interfere with the daily quality of life [30]. Treatments for ASD vary with the degree and nature of symptoms. Treatment can be behavioral, developmental, educational, social-relational, pharmacological, or psychological, and it can also be complementary. The type of treatment a child receives for ASD depends on the child's needs. Autism is a spectrum disorder (it presents differently, ranging from mild to severe), and each child with it is unique, therefore, there are various treatments [31]. Symptoms of ASD can sometimes overlap with some disorders, like ADHD. Consequently, treatment approaches should prioritize addressing an individual's needs rather than solely relying on the diagnostic label [32].

Behavioral and communication treatments can be deployed in discrete trial training, pivotal response training, early intensive behavioral intervention, and verbal and behavioral intervention. In a developmental method, the relationship-based approach goes in line, also known as Floortime, as it involves getting on the floor with the child to play and do the activities the child likes. A visual-based approach that employs a picture exchange communication system helps an ASD patient ask questions and communicate through special symbols [31].

As it stands, there is no medication to treat ASD, hence no cure for it. However, some medications can help with related symptoms. In some instances, medication can be beneficial in addressing various challenges associated with autism spectrum disorder (ASD). For instance, it may help manage symptoms like high energy levels, difficulty focusing, or self-harming behaviors such as head banging or hand biting. Medication can also effectively address co-occurring psychological conditions like anxiety or depression, as well as medical conditions such as seizures, sleep problems, or gastrointestinal issues [32].

Risperidone (Risperdal) and aripiprazole are the only United States Food and Drug Administration (FDA)-approved drugs for treating ASD in children. Risperidone may be prescribed for children aged five to 16 to manage irritability and aggression, while aripiprazole may be prescribed for children aged six to 17 [31].

Role of early intervention in improving outcomes in children with ASD

Dowsen et al. showed the efficacy of the early start of behavioral therapy in a randomized controlled trial to improve the onset of behavioral symptoms in children. Children between the ages of 18 and 30 months who received Early Start Denver Model (ESDM) intervention demonstrated significantly improved intelligence quotient (IQ), adaptive behavior, and autism diagnosis compared to those who received community intervention. After two years, the ESDM group showed an average improvement of 17.6 standard score points, while the comparison group improved by 7.0 points relative to their baseline scores. The ESDM group maintained a growth rate in adaptive behavior similar to typically developing children, while the comparison group showed greater delays in adaptive behavior over the same period. Additionally, children in the ESDM group were more likely to experience a change in diagnosis from autism to pervasive developmental disorder, not otherwise specified, compared to the comparison group [33]. An important term in ASD development is "sensitive periods" in human brain development. Sensitive periods refer to specific developmental phases during which the brain is particularly receptive to learning and acquiring particular skills, and how these skills can have a longer-lasting impact on human behavior when repetitively exposed. This is due to the brain’s ability to be highly stimulated in its early developmental phase. Penhune VB pointed out that during these sensitive periods, a child's learning is more susceptible to being affected by the external environment and frequently exposed stimuli than less frequently exposed stimuli [34].

Neuroplasticity refers to the brain's ability to reorganize and form new neural connections throughout life, which is especially pronounced during early childhood (two to three years old). When children receive early intervention for autism, they are exposed to various learning experiences that can modify and strengthen neural connections. This plasticity allows the brain to adapt and reorganize in response to therapeutic interventions [35]. By consistently stimulating the brain through appropriate interventions, early intervention can help shape neural pathways and improve cognitive and behavioral functioning in children with autism. Thus, early intervention leading to neuroplasticity can prevent ASD manifestations and modify their course [36]. Fuller EA et al. describe that major improvements were observed in children’s social communication when clinicians’ early intervention showed that ASD spectrum disorder prognosis could largely be prevented at the earliest stages of children’s communication development [37]. 

Challenges to the early diagnosis of autism spectrum disorder 

Identifying ASD at an early stage can present challenges due to various factors. Autism is a spectrum disorder with heterogeneous symptoms. Due to the unique traits and characteristics exhibited by each individual with autism, it is challenging to develop a universally comprehensive diagnostic test. This variability necessitates healthcare professionals to accurately and consistently interpret a wide range of behaviors and observations to make an accurate diagnosis. Also, ASD may occur with other medical conditions that make it difficult to know what is causing the differences in the child. Examples of these conditions or comorbidities are ADHD, language disorders, selective mutism, intellectual disability, and dyspraxia [38]. Many studies have also recognized the place of cultural and social effects on ASD diagnosis. Autism spectrum disorder is not limited to specific geographical regions, linguistic or cultural groups, or socio-economic backgrounds. It can affect individuals from diverse backgrounds, making it a condition that transcends geographical, cultural, and socio-economic boundaries. Autism spectrum disorder can be described as a disorder without borders. However, the availability of resources for identifying and treating ASD, as well as the attitudes of communities towards the disorder, vary significantly within and between different geographical regions, cultures, and socio-economic statuses (SES) [38].

Autism is still not fully understood, leading to a significant lack of knowledge and awareness. While early diagnosis is believed to offer better prospects for individuals with autism, it presents challenges in evaluating the effectiveness of interventions and the accuracy of the initial diagnosis [37]. Also, the absence of a conclusive genetic or medical test poses a challenge to achieving a definitive diagnosis of autism, even in older individuals. A recent pediatrician survey revealed that insufficient information is frequently available to accurately diagnose autism spectrum disorder (ASD) in children [38]. All assessments are subjective, and the reliance on behavioral observations and subjective judgments during assessments can introduce variability and subjectivity into the diagnostic process [37]. It has also been reported that some autism symptoms may not appear until later in childhood; it can be challenging to make an early diagnosis of ASD due to these gradual onset patterns of the disorder’s symptoms. Due to the variations in individual development, diagnosing autism at a very young age may not account for the normal fluctuations and differences in developmental rates among children. Furthermore, symptoms of autism can evolve and change over time, leading to fluctuations in their visibility and making specific symptoms more or less apparent as time progresses [37].

Future directions in the early diagnosis of ASD

Early diagnosis and intervention of ASD can significantly improve outcomes for individuals with ASD. Researchers are actively investigating the development of objective biomarkers that can be utilized in the early diagnosis of ASD. These biomarkers may encompass neuroimaging, epigenetic alterations, and other measurable physiological factors associated with ASD [39]. Early screening and diagnosis of ASD are critical, but current methods may not identify all cases early enough. Therefore, researchers are investigating new technologies and tools to quickly and accurately identify children at risk for ASD in infancy [40]. Research has found that the use of artificial intelligence and machine learning applications might be helpful in the diagnosis of ASD. Patterns in behavioral and physiological data are analyzed to provide an earlier and more accurate diagnosis of ASD [41].

Advances in assessment methods, including standardized test batteries encompassing multiple clinical domains, may lead to a more accurate diagnosis of ASD. New assessment methods include automated or computerized versions integrating cognitive testing, sensory testing, and neuropsychology testing [42]. These are just a few exciting directions in early diagnosis research for autism spectrum disorder. Improved and early diagnosis of ASD can lead to earlier and more effective interventions and ultimately contribute to an improved quality of life for individuals with the condition.

Strengths and limitations

This article has several strengths. It provides a comprehensive overview of ASD, covering various aspects such as definition, symptoms, prevalence, diagnostic tools, treatment options, and the significance of early intervention. Also, using references from reputable sources enhances the credibility of the information presented, as it is backed by scientific studies and data from reliable organizations like the Centers for Disease Control and Prevention (CDC). The article is well-written, clear, and concise, making it understandable to a broad audience. Technical terms are explained, and the information flows smoothly.

Additionally, the article includes a flowchart and a table to present screening guidelines and diagnostic criteria. This aids in simplifying complex information and improving reader comprehension. Moreover, the article references studies and data up to 2021, ensuring that the information provided is current and relevant to the field.

However, there are some limitations to the article. It lacks information about the author's credentials or affiliations, which could impact the perceived authority and expertise of the content. While it briefly discusses challenges in early diagnosis, a more in-depth analysis of potential drawbacks and controversies related to ASD diagnosis and early intervention would be beneficial. The article generalizes treatment options without highlighting that their effectiveness can vary depending on individual needs and symptom severity. Additionally, it does not address alternative perspectives, such as the neurodiversity model, which views autism as a natural neurological variation rather than a disorder.

Despite these limitations, the article serves as a helpful introductory overview of ASD, early diagnosis, and the importance of early intervention. However, readers are encouraged to seek additional sources and perspectives to develop a comprehensive understanding of the evolving field of autism spectrum disorder.

## Conclusions

Autism spectrum disorder is a complex neurodevelopmental condition characterized by challenges in social interaction, communication, and restricted or repetitive behaviors. Its prevalence has increased, highlighting the need for better understanding and early detection. Early diagnosis (by 18-24 months) is pivotal in improving outcomes for children with ASD. It provides opportunities for early interventions that can positively impact cognitive, language, and social-emotional functioning. Early Start Denver Model (ESDM) interventions have shown promising results in improving IQ, adaptive behavior, and autism diagnosis in young children.

However, the process of early diagnosis faces several challenges, including the heterogeneity of symptoms, comorbidities with other conditions, and the lack of universally comprehensive diagnostic tests. Additionally, cultural and social factors can influence the diagnosis and acceptance of ASD. Overdiagnosis and overtreatment are potential risks of early diagnosis, and healthcare professionals must carefully consider individual needs and avoid unnecessary interventions.

Early diagnosis of ASD benefits individuals with the condition and supports families in accessing appropriate services and reducing stress. It can lead to more targeted interventions, better social outcomes, and increased independence in adulthood. As we advance in understanding and identifying ASD, it is crucial to balance recognizing neurodiversity and providing timely and effective support for individuals with ASD and their families. By addressing these challenges and embracing new research, we can continue to improve the lives of those affected by ASD.
